# Divergent secular trends in blood pressure and body mass index in children and adolescents in Hong Kong

**DOI:** 10.1038/s41598-017-05133-2

**Published:** 2017-07-06

**Authors:** Man Ki Kwok, Gabriel M. Leung, Thomas W. H. Chung, Karen K. Y. Lee, C. Mary Schooling

**Affiliations:** 1 School of Public Health, Li Ka Shing Faculty of Medicine, The University of Hong Kong, Hong Kong Special Administrative Region, China; 2 Student Health Service, Department of Health, Hong Kong Special Administrative Region, China; 3 City University of New York Graduate School of Public Health and Health Policy, New York, United States

## Abstract

Secular trends in blood pressure (BP) and body mass index (BMI) during childhood and adolescence are sentinels for the future population cardiovascular disease burden. We examined trends in BP z-score (ages 9–18 years from 1999 to 2014) and BMI z-score (ages 6–18 years from 1996 to 2014) in Hong Kong, China. Overall, BP z-score fell, systolic BP from 0.08 to −0.01 in girls and from 0.31 to 0.25 in boys. However, the trends were not consistent, for both sexes, systolic BP z-score was stable from 1999, decreased slightly from 2002 to 2005 and increased slightly to 2014, diastolic BP z-score decreased slightly from 1999 to 2004 and then remained stable to 2014. In contrast, BMI z-score rose from −0.15 to −0.01 in girls and from 0.14 to 0.34 in boys, mainly during 1997 to 2010. The upper tail of the systolic (except boys) and diastolic BP distribution shifted downwards, whereas the entire BMI distribution shifted upward. BP declined slightly whereas BMI rose in Hong Kong children and adolescents during the last 20 years, with systolic BP and BMI in boys above the reference. This warrants dual action in tackling rising BMI and identifying favorable determinants of BP, particularly targeting boys.

## Introduction

Secular trends in blood pressure (BP) and body mass index (BMI) during childhood and adolescence are key global cardiovascular health surveillance indicators. In many long-term developed Western countries, adult systolic BP has decreased since the 1980s^1^, whereas adult BMI has monotonically increased^2^. These trends are congruent with declining haemorrhagic stroke but increasing ischemic stroke^3^ given lower BP particularly reduces haemorrhagic stroke^1^ while higher BMI promotes the atherosclerosis implicated in ischemic stroke^4^. In children and adolescents in long-term developed Western populations, declining BP has been documented since the mid-20^th^ century^5^, followed by a rise from the mid-1980s in the United Kingdom^6^ and from the early 1990s in the United States^7^. A monotonic rise in BMI has occurred since the 1960s^8^, suggesting trends in BP and BMI during childhood and adolescence foreshadow changes in patterns of adult cardiovascular diseases^5^.

Secular trends in cardiovascular disease, and its drivers, in non-Western settings are more varied. For example, over the last 20 years, BP has increased since the mid-1990s in China^9, 10^, but recently declined in the Seychelles^11^ and systolic, but not diastolic, BP has clearly declined in South Korea^12^. BMI has increased in China^13^, the Seychelles^11^, and also in South Korea^12^. In short, BMI has been generally increasing in children and adolescents in most non-Western populations. Conversely, with longer experience of economic transition, BP tends to decline, as in developed Western settings, but systolic and diastolic BP trends may differ^14^.

The observed contrasting trends in BMI and BP in early life with economic development could underlie and inform the observed contrasting trends in cardiovascular disease mortality, which are poorly understood^15^, however these contrasting trends could also be artifacts of sampling at different ages and developmental stages^16^, secular increases in height^14^, small samples and few time points^17^. Hong Kong is an ethnically homogeneous Chinese population with a different social and economic history from the rest of China^18^. Children and adolescents today in Hong Kong represent the first generation of Chinese to grow up in a post-industrial Chinese setting with living standards and social infrastructure similar to Western Europe, but a much more rapid trajectory of economic development^19^, which might also have consequences for current patterns of disease^20^. This raises the question as to whether trends in BP and BMI in Hong Kong would be similar to currently rapidly developing China, or other recently developed non-Western countries, or to Western countries with a longer history of economic development. To address this question, we examined trends in BP at ages 9–18 years from 1999 to 2014 and BMI at 6–18 years from 1996 to 2014 in Hong Kong, where population-representative information on BMI and BP for school age children and adolescents is available.

## Results

A total of 402,040 children and adolescents aged 9–18 years contributed to BP measurements from 1999 to 2014 and 1,898,816 aged 6–18 years contributed BMI measurements from 1996 to 2014. Table 1 shows mean age- and height-standardized systolic BP z-score decreased from 0.08 in 1999 to −0.01 in 2014 for girls and from 0.31 to 0.25 for boys. Diastolic BP decreased from −0.12 to −0.22 for girls and from −0.03 to −0.13 for boys. Concomitantly, the proportion with pre-hypertensive BP or hypertensive BP decreased from 18.3% to 9.9% in girls and from 30.6% to 26.4% in boys. In contrast, Table 2 shows mean age-standardized BMI z-score increased from −0.15 in 1996 to −0.01 in 2014 for girls and from 0.14 to 0.34 for boys. Correspondingly, the proportion of overweight or obesity also increased from 13.2% to 14.0% for girls and from 19.0% to 22.4% for boys. Table 3 shows the mean difference in age- and height-standardized systolic BP z-score was −0.006 per year in girls and −0.004 in boys and for diastolic BP was −0.002 in both sexes (model 1). The age-standardized BMI z-score was higher by 0.016 per year in girls and 0.022 in boys. Comparing the changes in BP z-score with BMI z-score over time (from an interaction term between BMI z-score and calendar year) in a regression of BP z-score on BM z-score, the positive coefficient for interaction suggests the association of BP z-score with BMI z-score was stronger over time (model 2).

**Table 1:** Age- and Height-Adjusted Systolic and Diastolic Blood Pressure (BP), Age-Standardized Systolic and Diastolic BP,^a^ Age- and Height-Standardized Systolic and Diastolic BP Z-scores^b^ and Proportion of Prehypertensive BP and Hypertensive BP^c^ Among 196,299 Boys and 205,741 Girls Aged 9 to 18 From 1999 to 2014 in Hong Kong.

Year	n	%	Age- and height-adjusted				Age- standardized				Age- and height-standardized				Prehypertensive BP	Hypertensive BP
			Systolic BP (mmHg)		Diastolic BP (mmHg)		Systolic BP (mmHg)		Diastolic BP (mmHg)		Systolic BP z-score		Diastolic BP z-score			
			Mean	SD	Mean	SD	Mean	SE	Mean	SE	Mean	SD	Mean	SD	%	%
**Boys**																
1999	9,001	4.59	110.5	8.2	61.0	2.6	110.1	0.4	61.9	0.2	0.31	1.04	−0.03	0.65	21.9	8.7
2000	12,073	6.15	109.6	8.1	60.7	2.5	110.2	0.3	61.3	0.2	0.37	1.05	−0.10	0.58	17.7	12.1
2001	11,778	6.00	109.5	7.9	60.7	2.4	110.6	0.3	60.9	0.2	0.35	1.08	−0.15	0.58	16.6	12.9
2002	12,471	6.35	109.3	7.8	60.6	2.4	110.6	0.3	61.1	0.2	0.35	1.06	−0.13	0.56	16.2	12.8
2003	11,070	5.64	109.5	7.9	60.6	2.4	109.6	0.4	60.4	0.2	0.24	1.09	−0.18	0.58	15.3	11.0
2004	12,357	6.29	109.6	7.8	60.7	2.4	108.7	0.3	60.0	0.2	0.16	1.04	−0.21	0.57	15.9	8.0
2005	12,406	6.32	109.5	7.7	60.6	2.4	108.0	0.4	59.7	0.2	0.12	1.01	−0.24	0.54	16.4	6.4
2006	13,224	6.74	110.0	7.7	60.8	2.4	109.1	0.3	60.7	0.2	0.17	0.99	−0.19	0.52	17.5	6.4
2007	13,408	6.83	110.1	7.7	60.8	2.4	108.9	0.4	60.5	0.2	0.20	1.01	−0.18	0.52	18.2	7.5
2008	13,309	6.78	110.5	7.6	60.9	2.3	109.1	0.3	60.6	0.2	0.18	1.03	−0.18	0.53	18.0	7.7
2009	12,896	6.57	110.1	7.5	60.8	2.3	109.8	0.3	60.9	0.2	0.22	1.04	−0.17	0.53	17.1	8.4
2010	10,248	5.22	109.6	7.2	60.7	2.2	109.6	0.4	61.0	0.2	0.22	1.03	−0.14	0.52	16.7	7.9
2011	13,418	6.84	111.1	7.7	61.1	2.4	109.8	0.3	60.9	0.2	0.26	1.05	−0.16	0.53	20.5	9.1
2012	13,221	6.74	111.3	7.8	61.2	2.4	110.4	0.3	60.9	0.2	0.29	1.04	−0.15	0.53	21.5	9.2
2013	14,291	7.28	110.9	7.7	61.1	2.4	110.3	0.3	61.4	0.2	0.28	1.05	−0.14	0.53	21.1	8.8
2014	11,128	5.67	110.7	7.5	61.0	2.3	108.8	0.4	61.0	0.2	0.25	1.03	−0.13	0.53	18.2	8.2
**Girls**																
1999	10,072	4.90	109.2	5.5	60.7	1.8	105.3	0.4	61.3	0.3	0.08	1.05	−0.12	0.69	12.4	5.9
2000	13,337	6.48	108.7	5.6	60.5	1.8	105.4	0.4	60.3	0.2	0.09	1.01	−0.19	0.62	10.4	5.5
2001	13,448	6.54	108.7	5.5	60.5	1.8	105.2	0.3	59.8	0.2	0.07	0.99	−0.23	0.58	9.3	5.2
2002	13,464	6.54	108.5	5.5	60.4	1.8	105.0	0.3	60.0	0.2	0.05	0.99	−0.23	0.58	9.7	4.5
2003	12,189	5.92	108.6	5.5	60.4	1.8	104.3	0.3	59.7	0.2	−0.02	0.97	−0.27	0.57	8.5	3.7
2004	13,552	6.59	108.6	5.5	60.5	1.8	103.2	0.3	59.2	0.2	−0.10	0.97	−0.30	0.59	7.0	2.9
2005	13,363	6.50	108.5	5.4	60.4	1.7	103.7	0.3	58.9	0.2	−0.11	0.93	−0.32	0.55	6.7	1.7
2006	13,788	6.70	108.7	5.4	60.4	1.7	103.9	0.3	59.5	0.2	−0.08	0.90	−0.27	0.53	7.0	1.3
2007	13,793	6.70	108.8	5.4	60.5	1.7	104.7	0.3	59.9	0.2	−0.03	0.92	−0.24	0.53	7.8	1.9
2008	13,717	6.67	109.0	5.3	60.6	1.7	103.7	0.3	59.5	0.2	−0.08	0.95	−0.27	0.54	7.4	1.8
2009	13,016	6.33	108.8	5.2	60.5	1.6	104.2	0.3	59.7	0.2	−0.02	0.96	−0.24	0.55	8.1	2.1
2010	9,817	4.77	108.3	5.1	60.3	1.6	103.9	0.4	59.7	0.2	−0.01	0.96	−0.22	0.55	8.2	2.5
2011	13,454	6.54	109.2	5.4	60.6	1.7	104.2	0.3	59.8	0.2	−0.04	0.96	−0.26	0.55	8.1	2.4
2012	13,350	6.49	109.4	5.3	60.7	1.7	104.2	0.3	59.8	0.2	−0.03	0.94	−0.25	0.53	8.1	2.1
2013	14,127	6.87	109.1	5.3	60.6	1.7	105.1	0.3	60.2	0.2	0.00	0.94	−0.23	0.53	7.8	2.3
2014	11,254	5.47	109.1	5.3	60.6	1.7	103.6	0.4	59.8	0.2	−0.01	0.95	−0.22	0.52	7.7	2.2

Abbreviations: BP: blood pressure, SD: standard deviation; SE: standard error; z-score: standard deviation score. ^a^Age-standardized systolic or diastolic BP relative to the 2000 World Health Organization World Population Standard. ^b^Mean systolic or diastolic BP in z-score relative to age-, sex- and height-standardized blood pressure standards from the United States National High Blood Pressure Education Group in 2004: 1 unit change in systolic BP z-score is approximated to 10.6 mmHg and 1 unit change in diastolic BP z-score is approximated to 11.3 mmHg. ^c^Prehypertensive BP was defined as systolic or diastolic blood pressure > = 90^th^ percentile but <95^th^ percentile or > = 120/80 mmHg; Hypertensive BP was defined as systolic or diastolic blood pressure > = 95^th^ percentile.

**Table 2:** Age-Adjusted Body Mass Index (BMI), Age-Standardized BMI,^a^ Age-Standardized BMI Z-scores^b^ and Proportion of Overweight and Obesity^c^ Among 957,577 Boys and 941,239 Girls Aged 6 to 18 From 1996 to 2014 in Hong Kong.

Year	n	%	Age-adjusted		Age-standardized		Age-standardized		Overweight	Obesity
			BMI (kg/m^2^)		BMI (kg/m^2^)		BMI z-score			
			Mean	SD	Mean	SE	Mean	SD	%	%
**Boys**										
1996	77,869	8.13	17.9	1.0	18.3	0.02	0.14	1.38	14.4	4.6
1997	86,890	9.07	18.9	1.6	18.4	0.01	−0.10	1.32	11.6	3.3
1998	57,492	6.00	18.4	1.5	18.5	0.01	0.06	1.36	13.5	4.3
1999	48,965	5.11	18.2	1.4	18.6	0.02	0.14	1.37	14.5	4.8
2000	47,379	4.95	18.2	1.3	18.6	0.02	0.18	1.37	15.0	4.9
2001	47,139	4.92	18.1	1.3	18.7	0.02	0.20	1.37	15.6	4.9
2002	49,116	5.13	18.1	1.3	18.6	0.02	0.19	1.38	15.2	5.1
2003	42,569	4.45	18.2	1.3	18.8	0.02	0.26	1.37	15.9	5.5
2004	45,208	4.72	18.2	1.3	18.8	0.02	0.27	1.37	16.4	5.5
2005	41,653	4.35	18.3	1.3	18.9	0.02	0.30	1.37	16.9	5.6
2006	42,709	4.46	18.3	1.3	18.9	0.02	0.28	1.39	17.0	5.8
2007	41,986	4.38	18.4	1.3	19.0	0.02	0.32	1.38	17.3	6.1
2008	42,254	4.41	18.4	1.3	19.1	0.02	0.37	1.39	17.8	6.8
2009	40,279	4.21	18.2	1.3	19.2	0.02	0.44	1.39	18.7	7.2
2010	34,047	3.56	18.1	1.3	19.2	0.03	0.43	1.40	18.4	7.5
2011	43,531	4.55	18.3	1.4	19.2	0.02	0.40	1.38	18.2	7.0
2012	48,459	5.06	18.1	1.5	19.1	0.02	0.35	1.37	17.3	6.6
2013	58,389	6.10	17.9	1.4	19.2	0.02	0.39	1.37	17.5	6.8
2014	61,643	6.44	17.6	1.3	19.0	0.02	0.34	1.37	15.9	6.5
**Girls**										
1996	70,970	7.54	17.9	1.1	17.9	0.02	−0.15	1.19	10.7	2.5
1997	94,507	10.04	19.2	1.6	18.1	0.01	−0.29	1.10	8.3	1.8
1998	59,055	6.27	18.7	1.6	18.1	0.01	−0.22	1.13	9.3	2.1
1999	47,903	5.09	18.5	1.5	18.1	0.01	−0.18	1.15	10.0	2.3
2000	46,968	4.99	18.4	1.5	18.1	0.01	−0.15	1.14	10.2	2.2
2001	46,558	4.95	18.3	1.4	18.1	0.01	−0.13	1.14	10.5	2.3
2002	47,929	5.09	18.3	1.4	18.1	0.01	−0.13	1.14	10.3	2.3
2003	42,440	4.51	18.4	1.4	18.2	0.02	−0.08	1.14	11.1	2.5
2004	44,391	4.72	18.4	1.4	18.3	0.02	−0.08	1.13	11.0	2.4
2005	42,043	4.47	18.4	1.4	18.3	0.02	−0.07	1.14	11.4	2.4
2006	41,915	4.45	18.5	1.4	18.3	0.02	−0.08	1.14	11.1	2.4
2007	41,219	4.38	18.5	1.4	18.4	0.02	−0.04	1.13	11.8	2.5
2008	41,226	4.38	18.5	1.4	18.5	0.02	0.01	1.13	11.9	2.8
2009	38,416	4.08	18.4	1.4	18.5	0.02	0.05	1.13	12.8	3.1
2010	32,410	3.44	18.2	1.3	18.5	0.02	0.06	1.16	13.4	3.3
2011	41,613	4.42	18.4	1.5	18.5	0.02	0.03	1.13	12.4	3.0
2012	46,945	4.99	18.2	1.5	18.5	0.02	−0.01	1.13	11.9	2.9
2013	56,496	6.00	18.0	1.5	18.5	0.02	0.02	1.12	12.0	2.9
2014	58,235	6.19	17.7	1.4	18.4	0.02	−0.01	1.11	11.1	2.9

Abbreviations: BMI: body mass index, SD: standard deviation; SE: standard error; z-score: standard deviation score. ^a^Age-standardized BMI relative to the 2000 World Health Organization World Population Standard. ^b^Mean BMI in z-score relative to the 2007 World Health Organization growth references for 5–19 years: 1 unit change in BMI z-score is approximated to 2.67 kg/m^2^. ^c^Overweight was defined using the International Obesity Task Force cut-offs as equivalent to an adult BMI of 25 or more; Obesity was defined as equivalent to an adult BMI of 30 or more.

**Table 3:** Mean Difference^a^ in Age- and Height-Standardized Systolic and Diastolic Blood Pressure (BP) Z-scores^b^ per Year Among Boys and Girls Aged 9 to 18 From 1999 to 2014 and Age-Standardized Body Mass Index (BMI) Z-scores^c^ per Year Among Boys and Girls Aged 6 to 18 From 1996 to 2014 in Hong Kong.

		No.	Model 1		No.	Model 2	
			β	95% CI		β	95% CI
**Boys**	Systolic BP z-score						
	Year	195649	−0.004	(−0.005, −0.003)	195633	−0.012	(−0.013, −0.011)
	BMI z-score *Year					0.004	(0.003, 0.004)
	Diastolic BP z-score						
	Year	205608	−0.002	(−0.002, −0.001)	205598	−0.003	(−0.004, −0.003)
	BMI z-score *Year					0.0004	(0.00002, 0.0008)
	BMI z-score						
	Year	957577	0.022	(0.021, 0.022)			
**Girls**	Systolic BP z-score						
	Year	205608	−0.006	(−0.006, −0.005)	205598	−0.013	(−0.013, −0.012)
	BMI z-score *Year					0.004	(0.003, 0.004)
	Diastolic BP z-score						
	Year	205608	−0.002	(−0.002, −0.001)	205598	−0.004	(−0.004, −0.003)
	BMI z-score *Year					0.001	(0.0006, 0.002)
	BMI z-score						
	Year	941239	0.016	(0.015, 0.016)			

Abbreviations: BP: blood pressure, BMI: body mass index, z-score: standard deviation score; β: beta coefficient. ^a^Model 1 was crude association. Model 2 additionally included BMI z-score time trend (i.e., interaction term between BMI z-score and Year). ^b^Mean systolic or diastolic BP in z-score relative to age-, sex- and height-standardized blood pressure standards from the United States National High Blood Pressure Education Group in 2004: 1 unit change in systolic BP z-score is approximated to 10.6 mmHg and 1 unit change in diastolic BP z-score is approximated to 11.3 mmHg. ^c^Mean BMI in z-score relative to the 2007 World Health Organization growth references for 5–19 years: 1 unit change in BMI z-score is approximated to 2.67 kg/m^2^.

**Figure 1:**
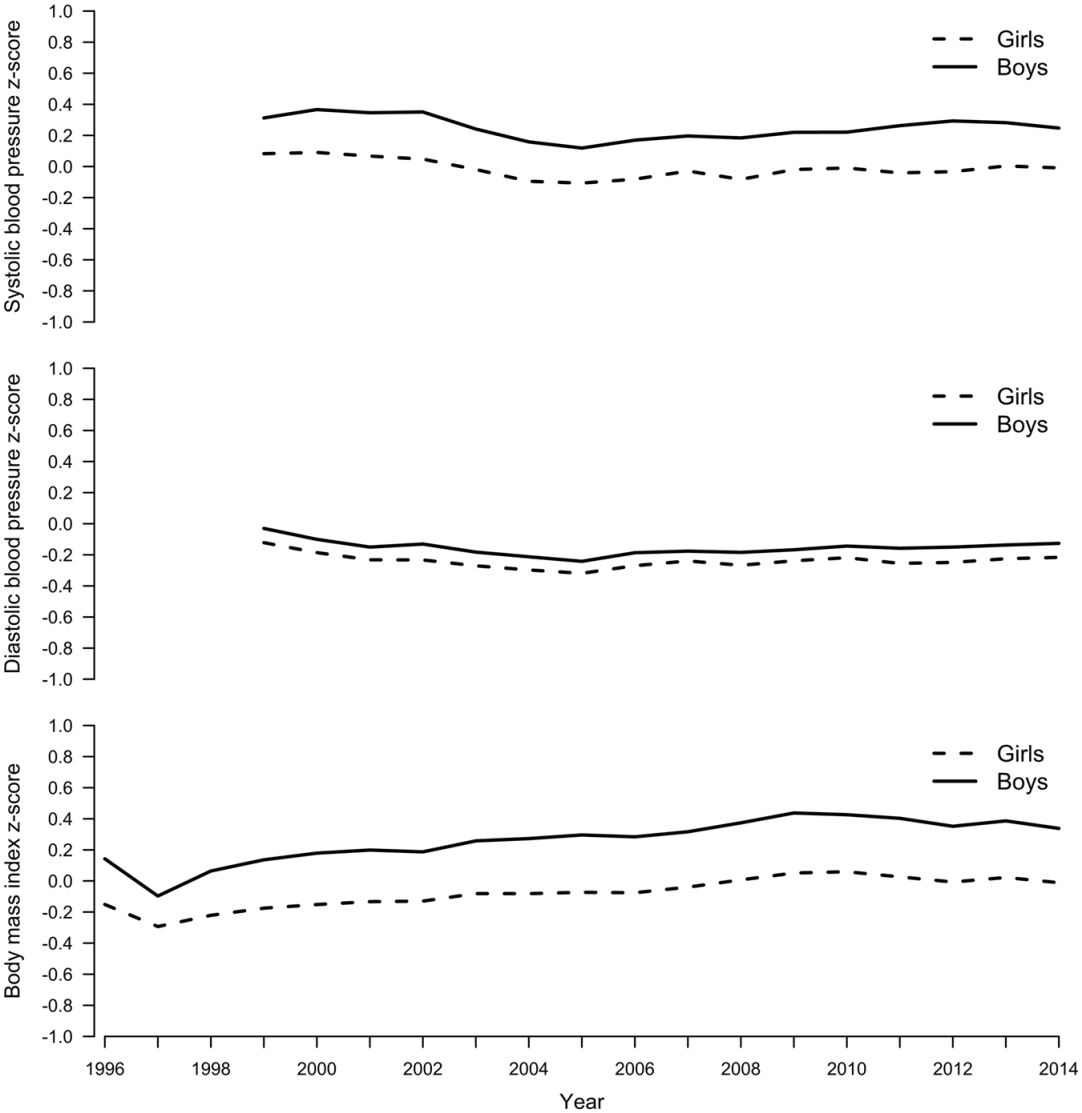
Mean Age- and Height-Standardized Systolic and Diastolic Blood Pressure (BP) Z-scores Among Boys and Girls Aged 9 to 18 From 1999 to 2014 and Mean Age-Standardized BMI Z-scores Among Boys and Girls Aged 6 to 18 From 1996 to 2014 in Hong Kong.

Figure 1 shows mean systolic BP z-score was relatively stable from 1999 and then decreased slightly from 2002 to 2005 followed by a slight increase afterwards to 2014 in both sexes. Mean diastolic BP had a slightly decreasing tendency from 1999 to 2004 and then remained stable to 2014. Conversely, mean BMI decreased from 1996 to 1997 and then increased until 2009/2010 followed by stagnation or a very slight decreasing tendency to 2014 in both sexes. Overall, boys had higher systolic BP and BMI than girls, but similar diastolic BP to girls. Somewhat similar changes were identified by sex-specific jointpoint analyses (Appendix Table 1), although no clear changes were identified for BMI in girls.

**Figure 2:**
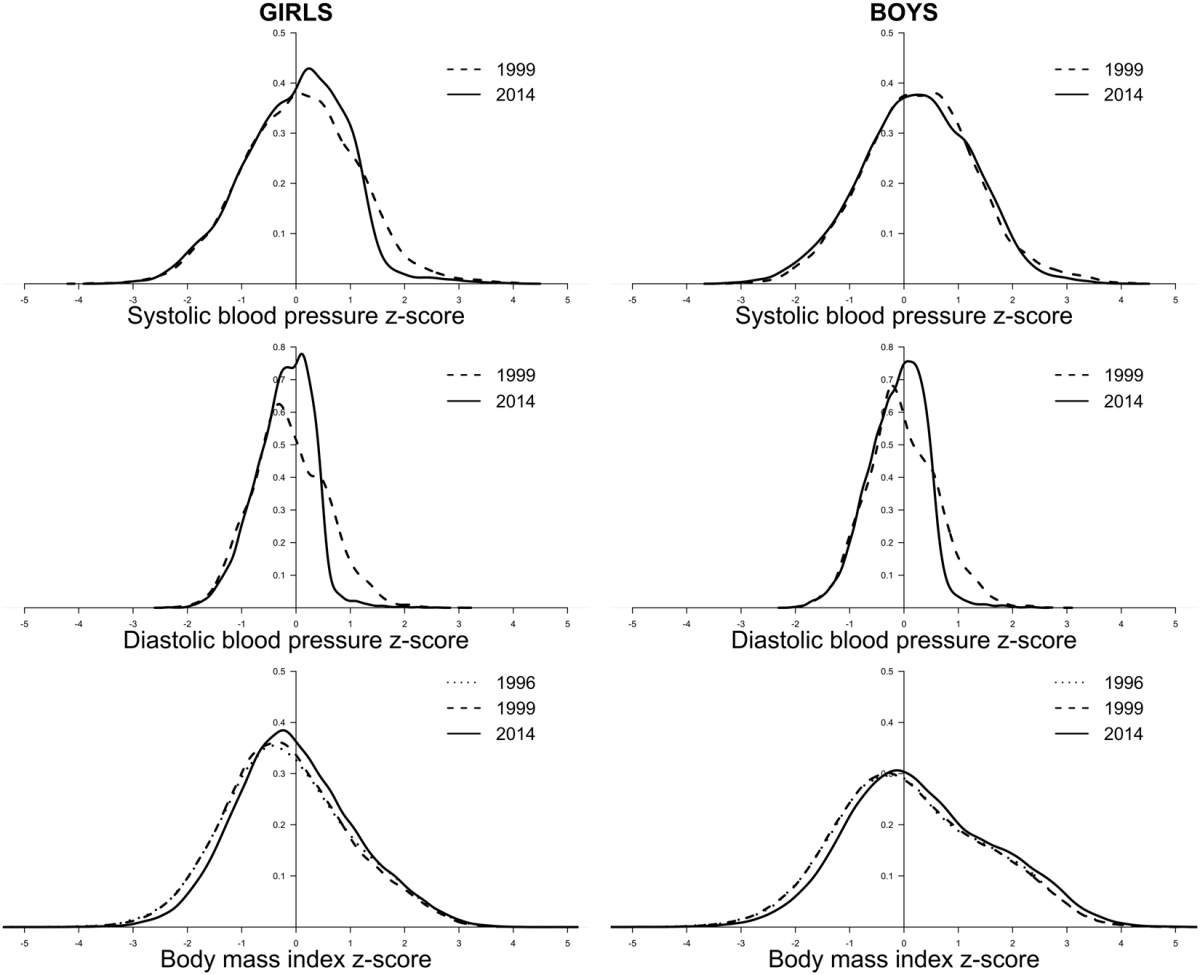
Distribution of Age- and Height-Standardized Systolic and Diastolic Blood Pressure (BP) Z-scores Among Boys and Girls Aged 9 to 18 in 1999 and 2014 and Distribution of Age-Standardized BMI z-scores Among Boys and Girls aged 6 to 18 in 1996 and 2014 in Hong Kong.

Figure 2 shows the distribution of systolic BP z-score had a marked downward shift in the upper tail for girls as did diastolic BP z-score in both sexes from 1999 to 2014, with overall a narrower distribution. The entire BMI z-score distribution shifted upward in both sexes from 1996 (or 1999) to 2014, but retained a right skewed distribution. The downward shift in the upper tail of the BP distribution and the upward shift of the entire BMI distribution was observed across all ages; except that the distribution of systolic BP in boys at age 12 years or above remained high from 1999 to 2014 (Appendix Fig. 1).

## Discussion

In a recently developed Chinese setting, overall systolic and diastolic BP declined modestly whereas BMI rose generally among children and adolescents over the last two decades. As a result by 2014, girls on average had BP and BMI below or close to the reference, whilst boys had systolic BP and BMI further above the reference. In addition, the upper end of the systolic and diastolic BP distributions shifted downward (except systolic BP in boys), whereas the entire BMI distribution shifted upward from 1996 to 2014.

This is the first large study with novel use of big data based on bi-annual BP and annual BMI measurements comprehensively covering childhood and adolescence, and so adds evidence concerning BP and BMI trends over nearly 20 years. To our knowledge, five previous studies examining both BP and BMI trends in children and adolescents over a similar period based on a population-representative sample from the same study have been published. The divergent patterns of BP and BMI in Hong Kong are inconsistent with patterns in rapidly developing China^9, 10, 13^, but are more similar to current trends in some other recently developed settings, such as the Seychelles^11^ and South Korea^12^, and are also similar to previous trends in long-term developed settings, such as the United Kingdom^6^ and the United States^7^. For instance, in recently developed South Korea, systolic BP z-score also declined but more strongly, by 0.08 per year in boys and 0.093 in girls from 1998 to 2008, with no clear changes in diastolic BP z-score. However, BMI increased from 1998 to 2005 before declining in 2007/08 (overall 0.033 per year) in boys with no changes in girls^12^, unlike our study showing similar BMI z-score trends in both sexes. Similarly, in earlier decades in the United States, where systolic BP slightly increased in 1979–1982 and then by 1993 gradually returned to same value as 1974, whereas diastolic BP z-score decreased by 0.009 per year in boys and 0.006 in girls from 1974 to 1993^21^. During the same period, BMI z-score monotonically increased by 0.028 per year in boys and 0.024 in girls^21^.

The BP and BMI profile appears to be more adverse in boys than girls, whether this is a reflection of cultural practises and pressures which emphasize different physical attributes in boys than girls is unknown^22^. Nevertheless, it does suggest that any future trends in cardiovascular disease presaged by these trends are going to be more adverse in men than women, and perhaps growing boys should particularly be targets of intervention. Notably, these sex differences are congruent with the sex differences in cardiovascular disease that widen with economic development^15^, suggesting that early life factors contribute to this sexual disparity.

Our findings are consistent with a narrower diastolic BP distribution and an upward shift in BMI distribution in South Korea^12^ and the Seychelles^11^, but are inconsistent with their downward shift in systolic BP in both sexes^11, 12^, given the downward shift in the upper tail of systolic BP was only observed in girls but not boys in our study. It is possible that factors which cause high BP in children and adolescents, such as renal parenchymal disease^23^, or risk factors for high BP such as sleep disordered breathing^24^ or early infections^25^, reduced over the period. The introduction of universal neonatal immunization for hepatitis B in Hong Kong in 1988^26^, or better access to care with the introduction of the Hospital Authority in Hong Kong in 1990^27^ could perhaps play a role, but leaves the lack of fall in the upper tail of the systolic BP distribution in boys aged 12 years or older unexplained. Systolic blood pressure rises more at puberty in boys than girls, and some coincidental factor may be offsetting the downward shift in pubertal boys, such as sleep disorders due to playing computer games^28^, or environmental factors that promote specifically testosterone^29^.

Overall, our study adds evidence of some downward secular trend in BP despite rising BMI. Higher BMI is a major risk factor for BP^30^, which has been confirmed in Mendelian randomization studies of adults^31^. We also found slightly stronger associations of BP with BMI over time, suggesting the trend of slightly increasing BP after the mid-2000s could be partly related to the increasing BMI trend which could have partly counteracted a potentially more pronounced downward BP trend. Nonetheless, differing trends for BP and BMI here and elsewhere^6, 7, 11, 12^ also suggest that other environmental factors affect BP. One possibility is sodium intake, because sodium restriction in infancy might affect blood pressure in adolescence^32^. In Hong Kong, the proportion of young children with sodium intake higher than the recommended intake is less than 10% at <18 months but 31% at 48 months^33^, most (60%) young children also have vegetable intake and fruit intake lower than the recommendations^33^. However, whether these intakes have changed over the relevant time period is not known. Moreover, the role of vegetables and fruit in BP has not been clearly established in trials^34^. Breastfeeding rates have increased since the 1990s in Hong Kong^35^, although whether breastfeeding affects BP has not been unequivocally established^36^. Smoking during pregnancy is very low (<5%) in Hong Kong^37^. Alternatively, physical inactivity is associated with lower lean mass and higher fat mass^38^. Both lean mass and fat mass are similarly positively associated with BP^39^. Also, an increasing trend in BMI may be mainly due to gain in body fatness given fat mass may have increased more than lean mass in Chinese children in recent decades^40^. As such, more gain in fat mass but less lean mass might be possible, but whether this could result in slightly decreasing BP while BMI is increasing remains elusive. Physical inactivity is prevalent in children and adolesents in Hong Kong and is not known to have changed in a similar way as BP in recent decades^41, 42^. A meta-analysis of short-term trials of physical activity did not reduce BP in children and adolescents^43^. Two small trials in obese children found physical activity modestly lowered BP, but one showed an increase in lean mass and no change in fat mass and weight^44^, while the other showed less abdominal fat and an increase in weight^45^. Future studies are needed to establish the causal role of physical activity and body composition (muscle mass and body fatness) in BP.

Several limitations are noted. First, attending the health assessment is voluntary. Differential selection by child health status or parental attributes or family socioeconomic position could bias the results, which is unlikely given health assessment at the Student Health Service is free and accessible to all public or private school students. However, it would only bias trends if the selection of children attending varied over time. Second, using a single BP measurement at a single visit may slightly overestimate average BP and pre-/hypertensive BP prevalence^46^, but would not affect comparisons over time. Third, random measurement error is possible, for which the large sample size compensates. Fourth, changes in devices over time could cause an artifactual change. Devices used to measure height and weight are reliable with little difference between devices. BP was measured by an automated oscillometric device, and re-checked with a sphygomanometer manually, which did not change during the period. Fifth, mid-arm circumference in children and adolescents may have increased in recent decades, implying an adult cuff size may be required for BP measurement^47^. However, BP was measured by nurses following a standard protocol with a cuff size appropriate to the age and size of the child. Sixth, we calculated BMI z-score using the 2007 WHO growth reference which used the same data as the 2000 United States Centers for Disease Control and Prevention reference but a different smoothing technique, and defined overweight and obesity using the International Obesity Task Force (IOTF) cut-offs which are based on nationally representative samples from six regions including Hong Kong^48^. As such, the absolute magnitude may not be directly comparable, but we focused on change for comparison across populations. Seventh, the study is descriptive and we can only speculate about the aetiologies of the observed divergent trends in BP and BMI. Finally, observed trends in BP might be driven by changes in potential risk factors for BP such as diet, physical activity and smoking, although most of these would also be expected to affect BP through BMI. The trends of these factors over the same period could not be analyzed due to lack of information.

In summary, as in some recently developed or long-term developed settings in earlier decades, divergent overall secular trends in BP and BMI were evident, resulting in systolic BP and BMI in boys further above the reference. Concerted efforts to tackle global increasing BMI trend is imperative; identifying the potential underlying factors that may alleviate the impact of rising BMI on BP or may lower BP independent of BMI are also important to improving future population cardiovascular health with a particular focus on boys.

## Methods

### Data Source

This study used routinely collected BP and BMI from the Student Health Service of the Department of Health, which provides free annual health assessments for all primary and secondary school students in Hong Kong^49^, for whom 9 years free universal public education (primary and 3-year junior secondary) has been provided since 1978 and 12 years (plus 3-year senior secondary) since 2008/09^50^. The Student Health Service was introduced in 1995/96 for primary school students, and was extended to secondary school students in 1996/97, but was suspended for secondary school students in year 2 and above in 2009/10 because of the Human Swine Influenza Vaccination Programme. The participation rate from 1995/96 to 2013/14 was 83.4%^51^. The health assessments include bi-annual assessments of BP (Primary 5 (age 10–11 years) onwards) and annual measurements of weight and height (Primary 1 (age 6–7 years) onwards). BP was measured by nurses on the right arm in a seated position after more than 10 minutes of rest with an age and size appropriate cuff size using an automated oscillometric device. Initial systolic or diastolic BP higher than the 90^th^ percentile for sex, age and height based on local references was re-checked by physicians with a sphygmomanometer after 15 minutes of rest and this second measurement was recorded. Height without shoes was measured by stadiometer to the nearest 0.1 centimetre and weight without shoes and outer clothing was measured by digital scales to the nearest 0.1 kilogram. BMI was calculated as weight in kilogram divided by height in meters squared. Coverage was incomplete in the early years, so we considered trends of BP since 1999 and BMI since 1996. We randomly selected one time point per participant so that there is no correlation between multiple measurements for the same participant. Given BMI was measured more often than BP and across a wider age range, more children and adolescents with BMI than BP were included.

### Outcomes

#### Blood pressure

Systolic and diastolic BP were considered as standard deviations (z-scores) relative to age-, sex- and height-standardized blood pressure standards from the 2004 United States National High Blood Pressure Education Group^52^, to account for compositional differences by age, sex and the secular trend in height. Prehypertensive BP was also considered, defined as systolic or diastolic BP greater than or equal to the 90^th^ percentile but less than the 95^th^ percentile based on age-, sex- and height-specific standards or 120/80 mmHg for both boys and girls. Finally, hypertensive BP was defined as systolic or diastolic BP greater than or equal to 95^th^ percentile^52^.

#### Body mass index

BMI was considered as z-scores relative to the 2007 World Health Organization (WHO) growth references for 5–19 years^53^. Overweight or obesity was also considered, defined as a BMI for age and sex corresponding to an adult BMI of ≥25 kg/m^2^ or ≥30 kg/m^2^ using the IOTF cut-offs^54^.

For completeness, age- and height-adjusted BP and age-adjusted BMI by sex were calculated as the internally weighted average BP and BMI accounting for differences in mean age and/or height across years within the sample. Age-standardized BP and BMI by sex were also calculated using the 2000 WHO World Standard Population for direct standardization^55^.

### Statistical analysis

To examine the secular trends in BP from 1999 to 2014 and BMI from 1996 to 2014, we plotted sex-specific mean BP and BMI z-scores by calendar year, and reported the average difference per year. We used jointpoint regression analysis with modified Bayesian information criterion to identify when the slope of BP and BMI z-scores trends changed significantly^56^. To identify changes in BP or BMI distribution, we plotted their distributions in 1996, 1999 and 2014 using a kernel density function. To identify at what ages changes in BP or BMI distribution occurred, we further plotted their distributions by each age in 1996, 1999 and 2014. We summarized the overall sex-specific changes in BP and BMI z-scores across years by regressing z-scores on calendar year using linear regression. We also assessed whether the strength (slope) of the association of BP with BMI changed over time by regressing BP z-score on BMI z-score, calendar year and the interaction term between BMI z-scores and calendar year, of which a positive interaction term coefficient indicates a stronger association of BP with BMI in later periods^11^. Statistical analyses were performed using Stata version 12.1 (Stata Corp, College station, Texas, USA), R version 3.0.1 (R Development Core Team, Vienna, Austria) and the jointpoint trend analysis version 4.2.0.1 (National Cancer Institute, USA)^57^.

### Ethics approval

The methods were carried out in accordance with the approved guidelines. The protocol was approved by the Health and Medical Research Fund, Government of the Hong Kong Special Administrative Region (SAR). The study obtained ethical approval from the University of Hong Kong-Hospital Authority Hong Kong West Cluster Joint Institutional Review Board. This study only used de-identified secondary data routinely collected by the Student Health Service, with informed consent obtained from a parent or guardian of the participants prior to participation to the voluntary health assessments.

## Electronic supplementary material

Appendix Table 1 & Figure 1
