# Supplementary material for: Divergent secular trends in blood pressure and body mass index in children and adolescents in Hong Kong
Source: Sci Rep. 2017 Jul 6;7:4763. doi: 10.1038/s41598-017-05133-2 (PMC5500554; doi:10.1038/s41598-017-05133-2)
Supplement: Supplementary file 1 — Appendix Table 1 & Figure 1 [file 41598_2017_5133_MOESM1_ESM.pdf]

Divergent secular trends in blood pressure and body mass index in children and adolescents in Hong Kong

Man Ki Kwok, Gabriel M Leung, Thomas WH Chung, Karen KY Lee, C Mary Schooling*

Appendix Table 1. Number of Jointpoints Identified for Trends of Mean Age- and Height-Standardized Systolic and Diastolic Blood Pressure (BP) Z-scores[a] Among Boys and Girls Aged 9 to 18 From 1999 to 2014 and Mean Age-Standardized BMI Z-scores[b] Among Boys and Girls Aged 6 to 18 From 1996 to 2014 in Hong Kong using Jointpoint Analyses with Modified Bayesian Information Criterion [c]

	Age- and Height-Standardized Systolic BP z-score		Age- and Height-Standardized Diastolic BP z-score		Age-Standardized BMI z-scores	
	Girls	Boys	Girls	Boys	Girls	Boys
Number of jointpoints	1	1	1	1	0	3
Years of jointpoints	**2005**	**2005**	**2004**	**2004**	**NA**	**1997, 1999, 2010**
Segmented periods	1999-2005	1999-2005	1999-2004	1999-2004	**NA**	1996-1997
	2005-2014	2005-2014	2004-2014	2004-2014		1997-1999
						1999-2010
						2010-2014

[a] Mean systolic or diastolic BP in z-score relative to age-, sex- and height-standardized blood pressure standards from the United States National High blood pressure Education Group in 2004: 1 unit change in systolic BP z-score is approximated to 10.6 mmHg and 1 unit change in diastolic BP z-score is approximated to 11.3 mmHg.

[b] Mean BMI in z-score relative to 2007 World Health Organization growth references for 5-19 years: 1 unit change in BMI z-score is approximated to 2.67 kg/m^2.

[c] Modified Bayesian Information Criterion (BIC) identifies the fitted model by penalizing extra parameters in model to avoid overfitting and can handle irregularities of likelihood function; lower modified BIC indicates better fitted model, with BIC decreases with reduced unexplained variation in dependent variable and fewer explanatory variables. The fitted model would be the parsimonious model with minimally sufficient number of jointpoints.

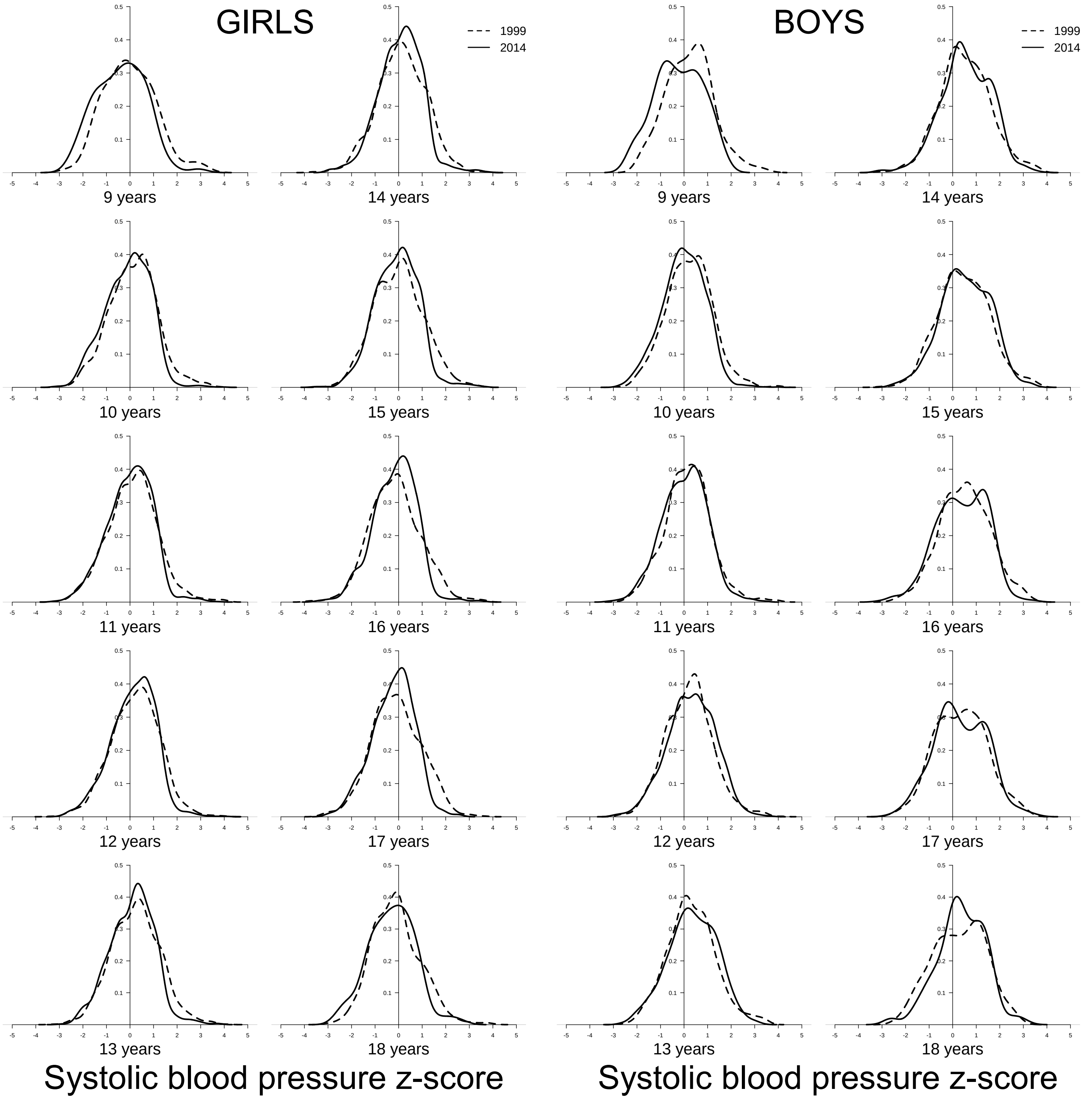

Appendix Figure 1. Distribution of Age- and Height-Standardized Systolic and Diastolic Blood Pressure (BP) Z-scores Among Boys and Girls at Each Age Group From 9 to 18 in 1999 and 2014 and Distribution of Age-Standardized Body Mass Index (BMI) Z-scores Among Boys and Girls at Each Age Group From 6 to 18 in 1996 and 2014 in Hong Kong

(To be continued)

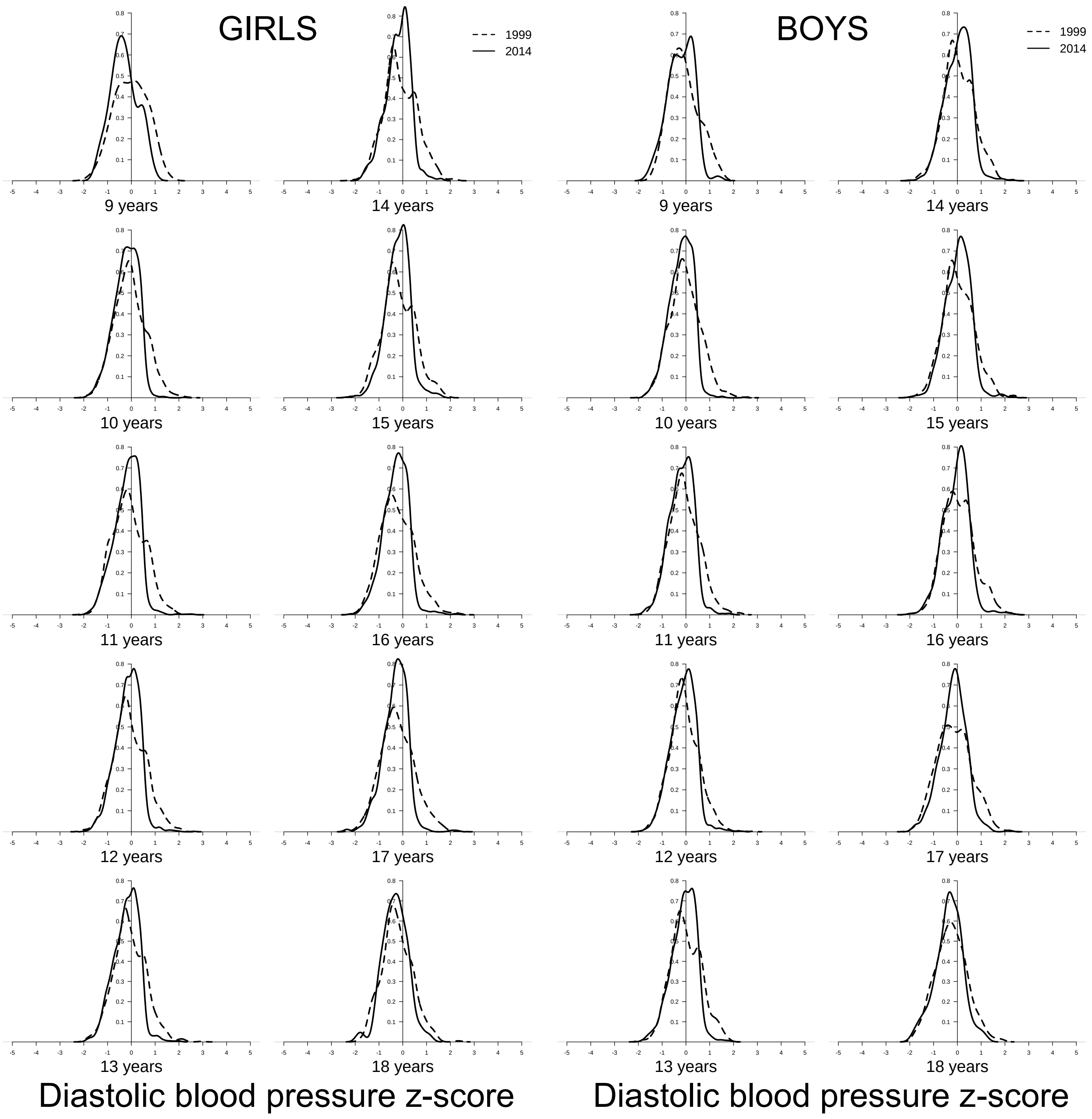

Appendix Figure 1. (continued)

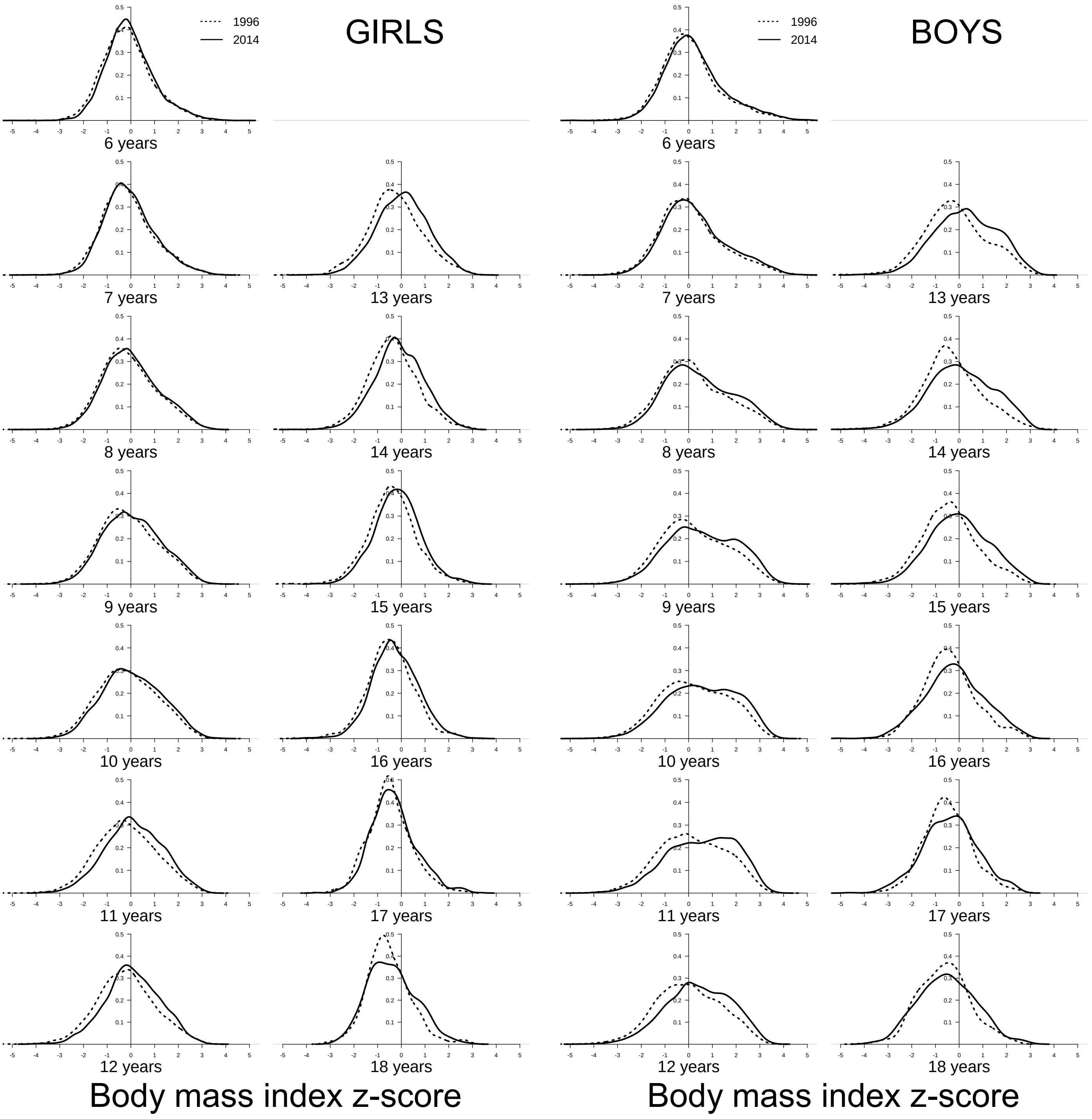

Appendix Figure 1. (continued)
